# β-Lapachone induces heart morphogenetic and functional defects by promoting the death of erythrocytes and the endocardium in zebrafish embryos

**DOI:** 10.1186/1423-0127-18-70

**Published:** 2011-09-22

**Authors:** Yi-Ting Wu, Che Yi Lin, Ming-Yuan Tsai, Yi-Hua Chen, Yu-Fen Lu, Chang-Jen Huang, Chao-Min Cheng, Sheng-Ping L Hwang

**Affiliations:** 1 Institute of Bioscience and Biotechnology, National Taiwan Ocean University, Keelung, Taiwan; 2 Graduate Institute of Life Sciences, National Defense Medical Center, National Defense University, Neihu, Taipei, Taiwan; 3 Institute of Cellular and Organismic Biology, Academia Sinica, Taipei, Taiwan; 4 Institute of Biological Chemistry, Academia Sinica, Taipei, Taiwan; 5 Institute of Nanoengineering and Microsystems, National Tsing Hua University, Hsinchu, Taiwan

**Keywords:** zebrafish, β-lapachone, heart morphogenesis, erythrocyte deficiency, endocardium, apoptosis

## Abstract

**Background:**

β-Lapachone has antitumor and wound healing-promoting activities. To address the potential influences of various chemicals on heart development of zebrafish embryos, we previously treated zebrafish embryos with chemicals from a Sigma LOPAC1280™ library and found several chemicals including β-lapachone that affected heart morphogenesis. In this study, we further evaluated the effects of β-lapachone on zebrafish embryonic heart development.

**Methods:**

Embryos were treated with β-lapachone or dimethyl sulfoxide (DMSO) at 24 or 48 hours post fertilization (hpf) for 4 h at 28°C. Heart looping and valve development was analyzed by whole-mount *in situ *hybridization and histological analysis. For fractional shortening and wall shear stress analyses, AB and Tg (*gata1*:*DsRed*) embryos were recorded for their heart pumping and blood cell circulations via time-lapse fluorescence microscopy. Dextran rhodamine dye injection into the tail reticular cells was used to analyze circulation. Reactive oxygen species (ROS) was analyzed by incubating embryos in 5-(and 6-)-chloromethyl-2',7'-dichloro-dihydrofluorescein diacetate (CM-H_2_DCFDA) and recorded using fluorescence microscopy. *o*-Dianisidine (ODA) staining and whole mount *in situ *hybridization were used to analyze erythrocytes. TUNEL assay was used to examine DNA fragmentation.

**Results:**

We observed a linear arrangement of the ventricle and atrium, bradycardia arrhythmia, reduced fractional shortening, circulation with a few or no erythrocytes, and pericardial edema in β-lapachone-treated 52-hpf embryos. Abnormal expression patterns of *cmlc2*, *nppa*, *BMP4*, *versican*, and *nfatc1*, and histological analyses showed defects in heart-looping and valve development of β-lapachone-treated embryos. ROS production was observed in erythrocytes and DNA fragmentation was detected in both erythrocytes and endocardium of β-lapachone-treated embryos. Reduction in wall shear stress was uncovered in β-lapachone-treated embryos. Co-treatment with the NQO1 inhibitor, dicoumarol, or the calcium chelator, BAPTA-AM, rescued the erythrocyte-deficiency in circulation and heart-looping defect phenotypes in β-lapachone-treated embryos. These results suggest that the induction of apoptosis of endocardium and erythrocytes by β-lapachone is mediated through an NQO1- and calcium-dependent pathway.

**Conclusions:**

The novel finding of this study is that β-lapachone affects heart morphogenesis and function through the induction of apoptosis of endocardium and erythrocytes. In addition, this study further demonstrates the importance of endocardium and hemodynamic forces on heart morphogenesis and contractile performance.

## Background

The heart is the first organ to form during vertebrate embryonic development. An embryonic heart tube is composed of outer myocardial and inner endocardial layers. After chamber formation, cardiac valves are formed from the endocardial cushion which is derived from the endocardium located at the atrioventricular boundary through an epithelial-to-mesenchymal transition [1]. The interaction between the myocardium and endocardium was shown to be important for developing a heart with normal functions. Bartman *et al*. demonstrated that reduced myocardial function can cause defects in endocardial cushion development via both *sih *and *cfk *zebrafish mutants associated with mutations in cardiac troponin T and a sarcomeric actin [2]. Similarly, a dysmorphic heart containing a compact ventricle and enlarged atrium with reduced contractility was observed in zebrafish *cloche *mutants with defects in the differentiation of all endothelial cells [3].

Blood circulation occurs early in the linear heart tube stage when diffuse oxygen is still sufficient to support various physiological processes, suggesting that blood circulation is required for heart morphogenesis [4]. The proper formation of a heart with normal functions is regulated by both a genetic program cascade and epigenetic factors (e.g., blood fluidic shear stresses) [5-7]. Fluidic shear stress is the frictional force derived from blood flow and plays an important role in embryonic vascular remodeling and cardiac morphogenesis [7-10]. In both *mlc2a*-null mice and *wea *zebrafish mutants (i.e., either a mutation in the *atrial myosin light chain 2 *or *atrial myosin heavy chain *gene), the mutation caused enlarged atria as well as a compact ventricle with underdeveloped trabeculae and a narrow lumen [11,12]. Since only atrial cardiomyoctyes completely lack myofibril organization, alteration of ventricle morphogenesis is likely attributable to changes in hemodynamic forces. Additionally, intracardiac fluidic forces were shown to be one of the essential factors for heart-looping and valve development in zebrafish embryos through blocking either the cardiac inflow or outflow by inserting glass beads [8].

β-Lapachone (3,4-dihydro-2,2-dimethyl-2H- naphthol[1,2-b] pyran-5,6-dione), a lipophilic ortho-naphthoquinone, was originally isolated from the lapacho tree (*Tabebuia avellanedae*) of South America [13]. β-Lapachone has antibacterial, antifungal, antiviral, anti-trypanosomal, and antitumor activities [14-18]. In a number of tumors (e.g., breast, colon, pancreatic, and lung cancers) with high expression levels of NAD(P)H:quinone oxidoreductase (NQO1), β-lapachone activates a novel apoptotic response [19-21]. In those tumors, NQO1 utilizes NAD(P)H as an electron donor to catalyze the two-electron reduction of β-lapachone to hydroquinone and a semiquinone intermediate in a futile cycle, resulting in the formation of reactive oxygen species (ROS) such as superoxide [21,22]. ROS can cause DNA damage, hyperactivation of poly(ADP-ribose) polymerase (PARP)-1 which depletes NAD^+ ^and ATP pools, that respectively result in an increase of the intracellular cytosolic Ca^2+ ^concentration, and activation of μ-calpain cysteine protease activity [23-25]. Treatment with dicoumarol (an NQO1 inhibitor) or BAPTA-AM (a Ca^2+ ^chelator) can inhibit cell death induced by β-lapachone [26,27].

In addition to being a model organism for probing vertebrate genetics and development, zebrafish have also proven to be ideal for screening small-molecule libraries to identify new therapeutic drugs [28,29]. To address the potential influences of various chemicals on the heart development of zebrafish embryos, we previously treated zebrafish embryos with chemicals from a Sigma LOPAC1280™ library and found several chemicals including β-lapachone that affected heart morphogenesis. In this study, we further evaluated the effects of β-lapachone on zebrafish embryonic heart development. We detected reduced fractional shortening, defects in heart-looping and valve development in β-lapachone-treated embryos. DNA fragmentation was also detected in both erythrocytes and the endocardium of β-lapachone-treated embryos. Furthermore, we demonstrated that the induction of apoptosis of the endocardium and erythrocytes by β-lapachone is mediated through an NQO1- and calcium-dependent pathway. This study, we believe, further demonstrates the importance of the endocardium and hemodynamic forces on heart morphogenesis and contractile performance in zebrafish embryos as a model system.

## Materials and methods

### Fish maintenance and collection of embryos

Adult zebrafish (*Danio rerio*) were raised at the zebrafish facility of the Institute of Cellular and Organismic Biology, Academia Sinica, Taipei, Taiwan. The fish were maintained in 20-L aquariums supplied with filtered fresh water and aeration under a 14-h light: 10-h dark photoperiod. Different developmental stages were determined based on previously described morphological criteria [30].

### β-Lapachone treatment

Embryos were treated with β-lapachone (Sigma-Aldrich, St. Louis, MO, USA) at a final concentration of 2 μM diluted with egg water at 24 or 48 hpf for 4 h at 28°C. Embryos from the same developmental stage treated with 0.2% DMSO for 4 h were used as the control.

### Whole-mount *in situ *hybridization

Whole-mount *in situ *hybridization was performed on embryos treated with 0.003% phenylthiocarbamide using digoxigenin-labeled antisense RNA probes and alkaline phosphatase-conjugated anti-digoxigenin antibodies as described previously [31]. Various templates were linearized, and antisense RNA probes were generated as follows: *BMP4 *(*Not *I/T7), *cmlc2 *(*Nco *I/SP6), *hbae1 *(*Not *I/T7), *nfatc1 *(*Spe *I/T7), *nppa *(*Nco *I/SP6), and *versican *(*Nco *I/SP6).

### *o*-Dianisidine (ODA) staining

Embryos were fixed with 4% paraformaldehyde overnight at 4°C. After several washes with PBST (1 × PBS and 0.1% Tween-20), β-lapachone- and DMSO-treated 48-hpf embryos were incubated in H_2_O_2 _(20 μl/ml)-activated ODA staining buffer (0.6 mg/ml ODA (Sigma-Aldrich, St. Louis, MO, USA) in 10 mM sodium acetate (pH 5.2) and 4% ethanol) for 15 min in the dark at room temperature and then washed with PBST several times.

### ROS assay

β-Lapachone- and DMSO-treated embryos were incubated in 5-(and 6-)-chloromethyl-2',7'-dichloro-dihydrofluorescein diacetate (CM-H_2_DCFDA) (Invitrogen, Carlsbad, CA, USA) at a final concentration of 500 ng/ml for 1 h in the dark at room temperature. After rinsing with egg water, embryos were examined using fluorescence microscopy equipped with a green fluorescent protein (GFP) filter.

### TUNEL assay and histological analysis

For the TUNEL assay to analyze apoptosis in the heart and erythrocytes, 48-hpf embryos were treated with either β-lapachone or DMSO for 4 h and then fixed at 52 hpf for ODA staining. Subsequently ODA-stained DMSO- and β-lapachone-treated 52-hpf embryos were fixed in 4% paraformaldehyde overnight at 4°C and embedded in paraffin according to standard procedures. Paraffin sections (5 μm) were dewaxed and rehydrated in PBST through an ethanol series. They were treated with 10 μg/ml proteinase K for 15 min at room temperature before DNA breaks were labeled with terminal deoxynucleotidyl transferase and fluorescein-dUTP according to protocols provided by the manufacturer (Roche Applied Bioscience, Mannheim, Germany). Nuclei were stained with DAPI. For the histological analysis, paraffin sectioning and hematoxylin (Vector, Burlingame, CA, USA) and eosin (Muto Pure Chemical, Tokyo, Japan) staining were performed according to standard procedures.

### Dextran rhodamine dye injection and photography

Diluted dextran rhodamine was injected into tail reticular cells of 48-hpf embryos using an IM300 microinjector (Narishigi, Tokyo, Japan). Images of embryos from various analyses were taken using an RT color digital camera (SPOT, Mchenry, IL, USA) on either a Zeiss Axioplan 2 microscope (Göttingen, Germany) or a Leica Z16 APO microscope (Wetzlar, Germany) equipped DIC or FITC mode.

### Wall shear stress and fractional shortening (FS) measurements

For wall shear stress and the FS analyses, 24-hpf Tg (*gata1*:*DsRed*) and 48-hpf AB embryos were treated with either β-lapachone or DMSO for 4 h, and then their blood cell circulations and heart pumping were recorded at 30 and 52 hpf, respectively, via time-lapse fluorescence microscopy using an AxioCam HRC camera with a high-speed recording mode (50 frames/s) under a Zeiss Axio Imager M1 microscope (Göttingen, Germany) with a tetramethylrhodamine isothiocyanate (TRITC; corresponding to pseudo-colored red) filter set.

### Real-Time Quantitative (Q) Reverse-Transcription (RT)-PCR

Q-RT-PCR was conducted and analyzed as described [32]. The primer pair for *nppa *was F-GGCAACAGAAGAGGCATCAGAG and R-GGAGCTGCTGCTTCCTCTCGGTC. The primer pair for *β-actin *was F-CCATTGGCAATGAGAGGTTCAG and R-TGATGGAGTTGAAAGTGGTCTCG.

## Results

### Phenotypes of β-lapachone-treated embryos

**Figure 1:**
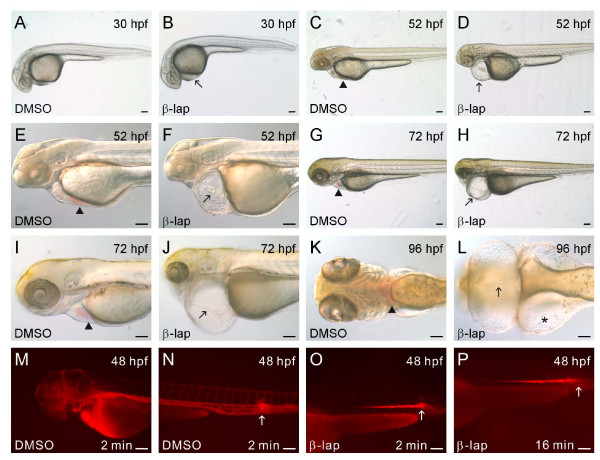
**Phenotype and circulation defect of β-lapachone-treated embryos**. Embryos at 24 hours post-fertilization (hpf) were treated with DMSO or β-lapachone for 4 h and examined at 30 (A, B), 52 (C-F), 72 (G-J), and 96 hpf (K, L). Dextran rhodamine dye-injected and DMSO-treated 48-hpf embryos 2 min after dye injection (M, N), and dextran rhodamine dye-injected β-lapachone-treated 48-hpf embryos 2 min (O) and 16 min after dye injection (P) are shown. Arrowheads indicate erythrocytes. The star indicates yolk edema. Black arrows point to the linear arrangement of the heart chambers and pericardial edema, while white arrows in panels N-P indicate dye injection sites. Scale bars represent 100 μm.

Embryos at 6 hpf were first treated with different concentrations (2, 5, 10, and 50 μM) of β-lapachone overnight; then the death of embryos was observed in all treatments (data not shown). Subsequently, 24-hpf embryos were treated with 2 μM β-lapachone for different time periods to evaluate its effect on heart development (data not shown). We then treated 24-hpf embryos with 2 μM β-lapachone for 4 h and found that β-lapachone-treated 30-hpf embryos showed pericardial edema compared to DMSO-treated embryos (Figure 1A & 1B). Pronounced pericardial edema accompanied by a linear arrangement of the ventricle and atrium which underwent bradycardia arrhythmia and the presence of only a few or no erythrocytes in the blood circulation were observed in β-lapachone-treated 52- and 72-hpf embryos (Figure 1C-J). Severe pericardial and yolk edema accompanied by the linear arrangement of the heart chambers and a lack of blood circulation were observed in β-lapachone-treated 96-hpf embryos compared to DMSO-treated embryos (Figure 1K & 1L). A blood circulation defect was further confirmed by injecting dextran rhodamine into both β-lapachone- and DMSO-treated 48-hpf embryos. The injected dextran rhodamine dye was readily observed in all vasculature of DMSO-treated embryos 2 min after the injection, while in β-lapachone-treated embryos, the dye remained in the yolk extension region close to the injection point for at least 16 min (Figure 1M-P).

### Defects in heart-looping and valve development and decreased cardiac output were recorded in β-lapachone-treated embryos

**Figure 2:**
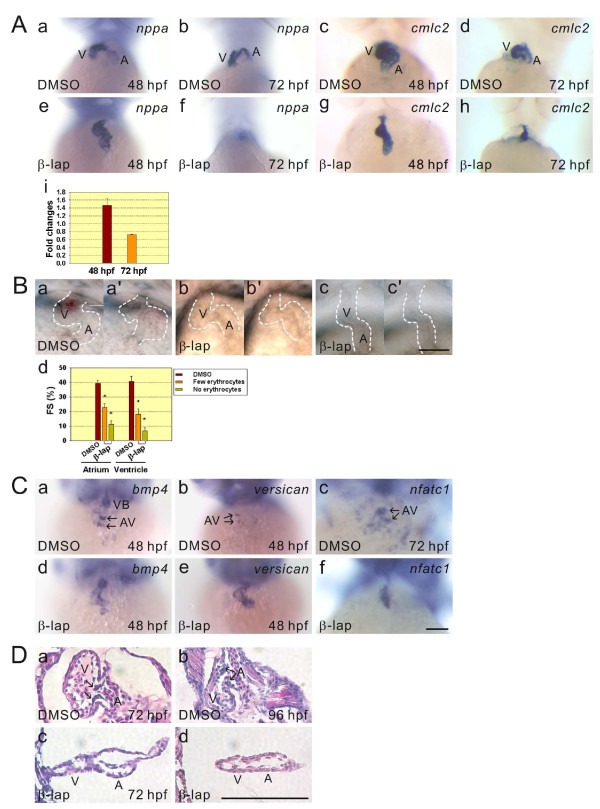
**Defects in heart-looping, valve formation, and contractile performance were detected in β-lapachone-treated embryos**. A: Embryos at 24 hours post-fertilization (hpf) were treated with DMSO or β-lapachone for 4 h and fixed at 48 and 72 hpf for *nppa *and *cmlc2 *hybridization (a-h). A Q-RT-PCR analysis indicated *nppa *expression levels in β-lapachone-treated 48- and 72-hpf embryos (i). B: Images of respective hearts with ventricles at either end-diastolic volume of DMSO (a), β-lapachone-treated embryo containing few erythrocytes (b), and β-lapachone-treated embryo containing no erythrocytes (c), or at end-systolic volume of DMSO (a'), β-lapachone-treated embryo containing few erythrocytes (b'), and β-lapachone-treated embryo containing no erythrocytes (c') are shown. Fractional shortening (FS) of the atrial and ventricular chamber of DMSO or β-lapachone-treated 52-hpf embryos was measured and calculated according to the formula, FS = (ED - ES)/ED × 100%, where ED is the end-diastolic diameter and ES is the end-systolic diameter of either the atrial or ventricular chambers (d). In β-lapachone-treated embryos, embryos containing few or no erythrocytes were recorded. Error bars indicate the standard error. Student's *t*-test was used to compare DMSO- and β-lapachone-treated embryos. * *p *< 0.001. C: DMSO- and β-lapachone-treated 48- and 72-hpf embryos were fixed for *bmp4*, *versican*, and *nfatc1 *hybridization. D: Paraffin sectioning and H&E staining of hearts of respective DMSO- and β-lapachone-treated 72- and 96-hpf embryos are shown. Arrows indicate the positions of the cardiac cushion (a) and valve (b). A, atrium; V, ventricle; VB, ventriculobulbal junction; AV, atrioventricular junction. Scale bars represent 100 μm.

To clarify the role of β-lapachone treatment in heart chamber morphogenesis, we treated 24-hpf embryos with either DMSO or β-lapachone for 4 h and then fixed them at 48 and 72 hpf for whole-mount *in situ *hybridization using *nppa *and *cmlc2 *as RNA probes. In DMSO-treated 48- and 72-hpf embryos, *nppa *expression was restricted to the outer curvature of the ventricle and atrium [10], whereas *nppa *was intensively expressed in the entire ventricle and atrium in β-lapachone-treated 48-hpf embryos (*n *> 50), and decreased *nppa *expression was later shown in β-lapachone-treated 72-hpf embryos (*n *> 50) (Figure 2A). Quantitative real time-RT-PCR analyses of the *nppa *expression level further confirmed the whole-mount *in situ *hybridization results (panel i in Figure 2A). In DMSO-treated 48- and 72-hpf embryos, *cmlc2 *was mainly expressed in looped ventricle and to some extent in the atrium, While, similar level of *cmlc2 *expression in the linearly arranged atrium and ventricle was found in β-lapachone-treated 48- and 72-hpf embryos (*n *> 50) (Figure 2A).

Furthermore, in order to evaluate the influence of β-lapachone treatment on cardiac output, we treated 48-hpf embryos with either DMSO or β-lapachone for 4 h and then recorded their heart pumping at 52 hpf (panel a-c' in Figure 2B). We grouped β-lapachone-treated embryos into either embryos containing few erythrocytes (*n *= 6) or no erythrocytes (*n *= 8). We found that the fractional shortening (FS) of both the atrium and ventricle was significantly decreased in both groups of β-lapachone-treated 52-hpf embryos compared to DMSO-treated embryos (*n *= 9) (panel d in Figure 2B).

During heart valve development, BMP signals from the myocardium promote the endocardial cushion to undergo the epithelial-mesenchymal transition [33,34]. Nfatc1 expression in the endocardial cushion is essential for its growth and subsequent valve remodeling [33]. Versican is an extracellular matrix component in the heart that is required for the development of endocardial cushion swelling [35]. Therefore, we selected *BMP4*, *versican*, and *nfatc1 *as RNA probes for whole-mount *in situ *hybridization to investigate heart valve development of zebrafish embryos treated with β-lapachone. We treated 24-hpf embryos with either DMSO or β-lapachone for 4 h and then fixed them at 48 and 72 hpf to perform whole-mount *in situ *hybridization. In DMSO-treated 48-hpf embryos, *BMP4 *was expressed in the myocardium located at the ventriculobulbal (VB) and atrioventricular (AV) junctions, whereas both *versican *and *nfatc1 *were mainly respectively expressed in the myocardium and endocardium at the AV junction of the heart in both DMSO-treated 48- and 72-hpf embryos (Figure 2C). Ectopic expressions of *BMP4 *(*n *> 50), *versican *(*n *> 50), and *nfatc1 *(*n *> 50) in both the ventricle and atrium were observed in β-lapachone-treated 48- and 72-hpf embryos compared to DMSO-treated control embryos (Figure 2C). Histological analyses further demonstrated the respective impairment of endocardial cushion formation and valve development in β-lapachone-treated 72- (*N *= 4, *n *= 20) and 96-hpf (*N *= 4, *n *= 20) embryos (Figure 2D). These results indicate that heart morphogenesis including heart-looping, formation of the heart chamber curvature and valve, and cardiac output were affected by β-lapachone treatment.

### β-Lapachone treatment induces the death of erythrocytes and endocardium

**Figure 3:**
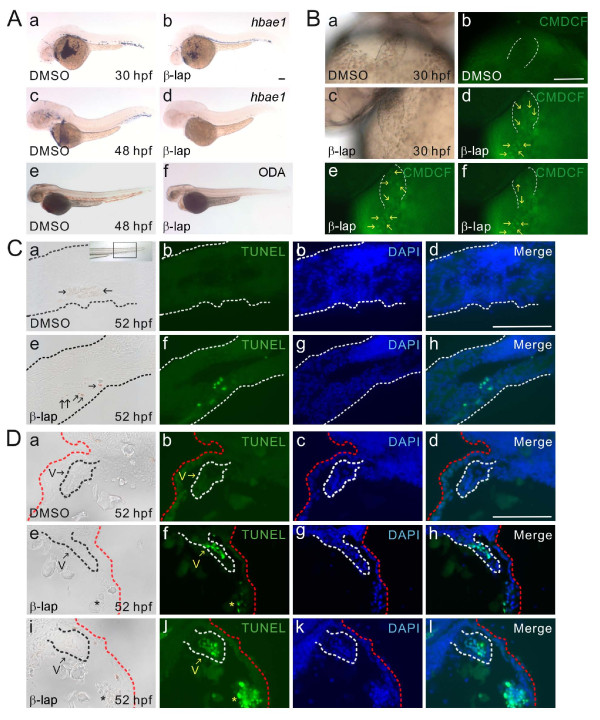
**Induction of ROS and DNA fragmentation in erythrocytes and the endocardium by β-lapachone treatment**. A: Embryos at 24 hours post-fertilization (hpf) were treated with DMSO or β-lapachone for 4 h and fixed at 30 and 48 hpf for either *hbae1 *hybridization or *o*-dianisidine (ODA) staining. B: DMSO- and β-lapachone-treated embryos were incubated with CM-H_2_DCFDA for 1 h at 29 hpf, and both bright-field (a, c) and fluorescent (b, d-f) images under a green fluorescent protein (GFP) filter were recorded. Atrial boundary is depicted by white dotted lines. Arrows indicate flowing erythrocytes with green fluorescence from the common cardinal vein to the atrium of the heart (d-f) of β-lapachone-treated embryos. C: Embryos were treated with DMSO or β-lapachone at 48 hpf for 4 h, fixed at 52 hpf, and stained with ODA. After paraffin sectioning, TUNEL reactions were conducted, and fluorescein-dUTP-labeled erythrocytes were detected in β-lapachone-treated embryos (f, h). DIC images are shown (a, e), and the inset figure in panel a shows the position of sectioning. Tail border is illustrated by white dotted lines. Arrows in panels a and e indicate ODA-stained erythrocytes. D: Embryos were treated with DMSO or β-lapachone at 48 hpf for 4 h and fixed at 52 hpf. After paraffin sectioning, TUNEL reactions were conducted, and fluorescein-dUTP-labeled cells located in the endocardium were detected in β-lapachone-treated embryos (f, h, j, l). In addition, fluorescein-dUTP-labeled erythrocytes were detected in the yolk near the heart (f, j). Red dotted lines indicate borders of head and yolk while white dotted lines illustrate ventricle boundaries. * indicates erythrocytes. V, ventricle. Scale bars represent 100 μm.

Embryos at 24 hpf were treated with either DMSO or β-lapachone for 4 h and then fixed at 30 and 48 hpf to analyze the presence of erythrocytes by whole-mount *in situ *hybridization using *hbae1 *(*hemoglobin *α *embryonic 1*) as a probe and hemoglobin staining with ODA (Figure 3A). In DMSO-treated 30- and 48-hpf embryos, *hbae1*-hybridized erythrocytes were readily observed in the dorsal aorta, posterior cardinal vein, and common cardinal vein. Relatively few *hbae1*-hybridized erythrocytes were observed in β-lapachone-treated 30-hpf embryos (*n *> 50), and no *hbae1 *hybridization was detected in β-lapachone-treated 48-hpf embryos (*n *> 50) (Figure 3A). Similarly ODA-stained hemoglobin was readily observed in both the dorsal aorta and posterior cardinal vein of DMSO-treated 48-hpf embryos; however, no ODA-stained hemoglobin was detected in β-lapachone-treated embryos (*n *> 50) (Figure 3A). Quantitative real time-RT-PCR analyses further confirmed the presence of very low expression levels of both *hbae1 *and *hbbe1 *(*hemoglobin β embryonic 1*) in β-lapachone-treated 48-hpf embryos (data not shown).

Since proerythroblasts from the intermediate cell mass expressing embryonic globins began to enter the circulation at 24 hpf, and hematopoiesis shifted from a primitive to a definitive wave at around 30 hpf, we also analyzed these two transcriptional factors which are important for definitive hematopoiesis [36]. Similar expression patterns and levels of *c-myb *(*n *> 50) and *ikaros *(*n *> 50) were found in DMSO- and β-lapachone-treated 30-hpf embryos, indicating that definitive hematopoiesis was not affected by β-lapachone treatment (Additional file, Fig. S1).

Since the incubation of β-lapachone with *Trypanosoma cruzi *preferentially causes the inhibition of DNA synthesis and DNA damage from the generation of oxygen radicals [15], we also evaluated whether ROS were generated in β-lapachone-treated embryos [37]. In the presence of peroxyl radicals (a type of ROS), CM-H_2_DCFDA is converted to the fluorescent dichlorofluorescein (DCF). We treated 24-hpf embryos with either DMSO or β-lapachone for 4 h and then incubated them with CM-H_2_DCFDA for 1 h at 29 hpf. Erythrocytes with green fluorescence were detected flowing from the common cardinal vein to the atrium of β-lapachone-treated 30-hpf embryos (*N *= 4, *n *> 50) but not in the respective DMSO-treated control embryos (Figure 3B).

Unlike mammals, zebrafish erythrocytes possess nuclei. Therefore, we treated 48-hpf embryos with either DMSO or β-lapachone for 4 h and fixed them at 52 hpf for ODA staining. These ODA-stained embryos were then used for the TUNEL assay. In DMSO-treated control embryos, a group of ODA-stained erythrocytes were detected in the tail blood island but fail to be labeled by fluorescein-dUTP (panel a-d in Figure 3C). In contrast, fluorescein-dUTP-labeled erythrocytes were found close to several ODA-stained erythrocytes in the tail blood island of β-lapachone-treated 52-hpf embryos (panel e-h in Figure 3C; *N *= 3, *n *= 15). However, ODA-stained erythrocytes did not show DNA fragmentation, indicating that fluorescein-dUTP-labeled erythrocytes had already lost their hemoglobin in the cytoplasm.

Although we did not detect ROS in the hearts of β-lapachone-treated 30-hpf embryos incubated with CM-H_2_DCFDA, TUNEL staining of paraffin sections revealed the presence of fluorescein-dUTP-labeled cells in the endocardium of the atrium and ventricle of β-lapachone-treated 52-hpf embryos (*N *= 3, *n *= 17), but not in DMSO-treated control embryos (Figure 3D). In addition, fluorescein-dUTP-labeled erythrocytes were observed in the yolk near the heart probably in the common cardinal vein (panels f & j in Figure 3D). These results indicated that β-lapachone treatment caused the occurrence of ROS in erythrocytes and DNA fragmentation in the endocardium and erythrocytes of zebrafish embryos.

### Decreased wall shear stress was identified in β-lapachone-treated embryos

**Figure 4:**
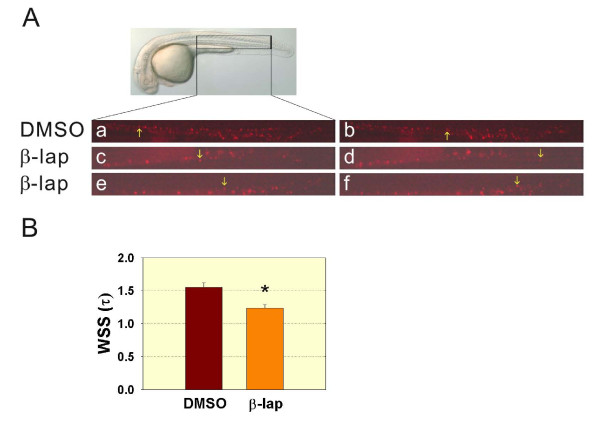
**Decreased wall shear stress was detected in β-lapachone-treated embryos**. Tg(*gata1*:*DsRed*) was treated with DMSO or β-lapachone for 4 h at 24 h post-fertilization (hpf), and blood cell circulation was recorded at 30 hpf. A: A DIC image of 30-hpf embryos is shown. The inset figure in panel A indicates the recorded region, and DsRed-labeled erythrocytes were recorded under the TRITC mode (a-f). Images corresponding to an individual DsRed-labeled erythrocyte (arrow) at time 0 and after traveling for some distance at time t are shown for respective DMSO-treated (a, b) and 2 representative β-lapachone-treated (c, d; e, f) embryos. B: The relative wall shear stress was calculated based on τ = 4 μQ/πR^3 ^where μ is viscosity, Q is the flow rate, and R is the blood vessel diameter. Student's *t*-test was used to compare DMSO- and β-lapachone-treated embryos. * *p *< 0.01.

Both the velocity gradient and blood viscosity when blood flows in a blood vessel are major physical factors determining the fluidic shear stress, and one of the major factors influencing the blood viscosity is hematocrit [38,39]. Since we observed a decreased number of erythrocytes in β-lapachone-treated embryos, we then evaluated the effect of β-lapachone treatment on the wall shear stress in the caudal artery of embryos. We treated 24-hpf Tg(*gata1*:*DsRed*) embryos with β-lapachone for 4 h and recorded the blood cell circulation at 30 hpf. Here, we used the formula 4 μQ/πR^3 ^to determine the wall shear stress, where μ is viscosity, Q is the flow rate, and R is the blood vessel diameter [7]. We first measured the average diameter of the caudal artery in DMSO- (20 μm) and β-lapachone-treated embryos (17 μm) and obtained the velocity of several individual DsRed-labeled erythrocyte in the caudal artery of DMSO- and β-lapachone-treated embryos by tracing the distance they traveled in a fixed period of time via time-lapse fluorescence microscopy. Flow rate (Q) was then calculated based on the average diameter of the caudal artery and average velocity of erythrocytes in both DMSO- (0.000533 μm/s) and β-lapachone-treated (0.000329 μm/s) embryos. We then estimated the relative shear stress using the viscosity value (0.008 Nt·s/m^2^) measured in oxygenated trout cannula blood at 26% hematocrit and a 90/s shear rate [40]. As shown in Figure 4, the wall shear stress (τ) significantly decreased in β-lapachone-treated (1.24 Nt/m^2^) 30-hpf embryos (*n *= 14) compared to DMSO-treated (1.55 Nt/m^2^) control embryos (*n *= 10).

### Co-treatment with either dicoumarol or BAPTA-AM and β-lapachone rescued both the heart-looping defect and erythrocyte-deficiency in circulation phenotypes

**Figure 5:**
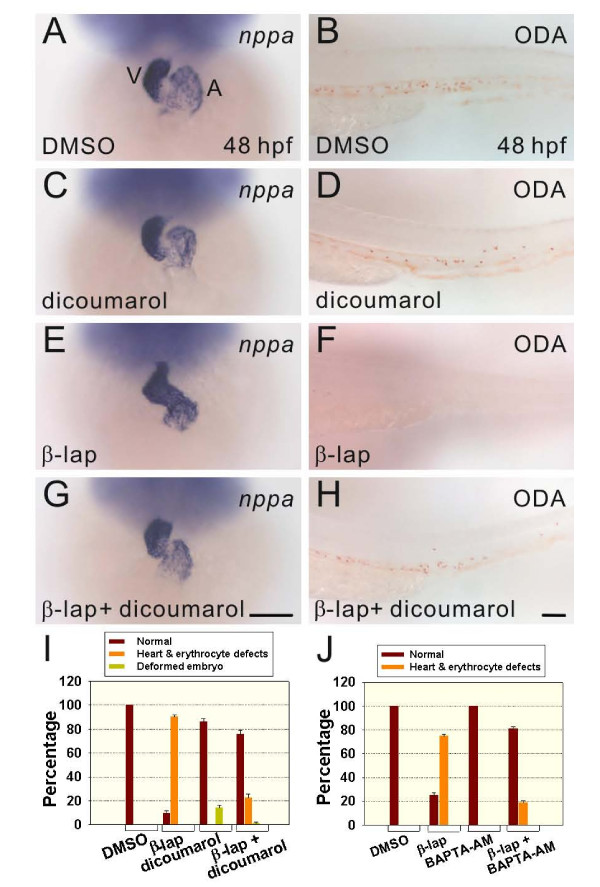
**Both dicoumarol and BAPTA-AM rescued the erythrocyte-deficiency in circulation and heart-looping defect phenotypes in β-lapachone-treated embryos**. Embryos at 24 hours post-fertilization (hpf) were treated with either 0.2% DMSO (A, B), 5 μM dicoumarol (C, D), 2 μM β-lapachone (E, F), or 2 μM β-lapachone and 5 μM dicoumarol (G, H) for 4 h and fixed for *nppa *hybridization and *o*-dianisidine (ODA) staining at 48 hpf. Higher-magnification ODA-stained images of the ventral tail region are shown (B, D, F, H). (I, J) Evaluation of the rescue effects by dicoumarol or BAPTA-AM. Error bars indicate the standard error. Scale bars represent 100 μm.

Since β-lapachone treatment induces NQO1- and μ-calpain-mediated apoptosis in a number of tumors [26,27], we evaluated whether the erythrocyte-deficiency in circulation and heart-looping defect phenotypes in zebrafish embryos induced by β-lapachone also adopted a similar mechanism. Dicoumarol, an inhibitor of NQO1, and BAPTA-AM, a Ca^2+ ^ion chelator which can affect the activity of calcium-dependent μ-calpain, were used [41,24]. We treated 24-hpf embryos with either 2 μM β-lapachone (*n *= 318 and 627) alone or together with either 5 μM dicoumarol (*n *= 215) or 50 μM BAPTA-AM (*n *= 557) for 4 h and fixed them at 48 hpf. In β-lapachone-treated 48-hpf embryos, a linear arrangement of the ventricle and atrium, ectopic *nppa *expression, and no ODA-stained erythrocytes were observed compared to DMSO- (*n *= 243) and dicoumarol-treated (*n *= 193) control embryos (Figure 5A-F). However, approximately 76% of 48-hpf embryos treated with both β-lapachone and dicoumarol showed levels of ODA-stained erythrocytes in circulation and a normal heart chamber morphology as assayed by *nppa *expression patterns, compared to those of control embryos (Figure 5G-I). Similarly, co-treatment with BAPTA-AM also rescued the β-lapachone-induced heart-looping defect and erythrocyte-deficiency in circulation phenotypes (data not shown). Approximately 81% of 48-hpf embryos treated with both β-lapachone and BAPTA-AM exhibited levels of ODA-stained erythrocytes in circulation and a normal heart chamber morphology, compared to DMSO- (*n *= 216) and BAPTA-AM-treated (*n *= 239) control embryos (Figure 5J). Collectively, these results indicate that the heart-looping defect and erythrocyte-deficiency in circulation phenotypes observed in β-lapachone-treated embryos were both induced by an NQO1- and calcium-mediated pathway.

## Discussion

β-Lapachone is currently in phase II clinical trials for treating pancreatic adenocarcinomas due to its antitumor activity [42]. It was also shown to have potential therapeutic use due to its wound healing-promoting activity [43]. In our study, we evaluated the potential effects of β-lapachone on heart development using zebrafish as the model organism.

### β-Lapachone treatment induces apoptosis of the endocardium and erythrocytes which in turn affect heart morphogenetic development and function

We observed a linear arrangement of the ventricle and atrium, bradycardia arrhythmia, reduced FS, and circulation with only a few or no blood cells in β-lapachone-treated 48-hpf embryos (Figure 1). Abnormal expression patterns of *cmlc2*, *nappa*, *BMP4*, *versican*, and *nfatc1 *and histological analyses demonstrated that there were defects in heart-looping and valve development in β-lapachone-treated embryos (Figure 2). Furthermore, we detected the presence of ROS in erythrocytes and DNA fragmentation in both the endocardium and erythrocytes of β-lapachone-treated embryos (Figure 3). These results indicate that defects in heart morphogenetic development and function can be attributed to apoptosis of the endocardium and erythrocytes induced by β-lapachone treatment.

An integral endocardium is essential for cardiac valve development because it gives rise to the endocardial cushion through an epithelial-to-mesenchymal transition process [33]. In addition, integral cardiac endocardial and myocardial interaction is required for proper heart development and contractile function [44]. Neuregulin-1 is a paracrine factor produced by endocardium endothelial cells. Mice possessing a homozygous mutation in *Neuregulin-1 *showed a heart that lacked trabecular formation in the ventricular wall, and slower contractions of the atrium and ventricle [45]. Similarly, Neuregulin/ErbB signaling was shown to be essential for ventricle trabeculation in zebrafish embryos, and knockdown *Neuregulin-1 *expression in zebrafish also resulted in heart development defects especially in the conducting system [46,47]. Therefore, our results further demonstrate the importance of the endocardium in cardiac structural and functional development.

As hematocrit influences the blood viscosity, which is one of the main physical factors affecting blood fluidic shear stress on the blood vessel wall [7,39], our relative wall shear stress approach also indicated a significant decrease in the wall shear stress in β-lapachone-treated embryos due to relatively few erythrocytes in the circulation (Figure 4). A decreased wall shear stress produced decreased hemodynamic forces which further impacted heart development and function in β-lapachone-treated embryos.

### Both the endothelium and erythrocytes are regulated by oxidative stress

Oxidative stress was shown to regulate the growth, survival, and apoptosis of vascular smooth muscle and endothelial cells [48]. Cytokines and growth factors (e.g., tumor necrosis factor-α, interleukin 1β, and angiotensin II) were shown to promote the generation of superoxide in endothelial cells by activating NADPH oxidase [49,50]. ROS then cause the release of cytochrome C from mitochondria to activate caspase, resulting in phosphatidylserine exposure, DNA fragmentation, and cell morphological changes [51]. A study on human umbilical vein endothelial cells further demonstrated that the activities of both caspase 3 and c-Jun N-terminal kinases (JNKs) were stimulated due to an increase in intracellular ROS levels [52].

The function and fate of erythrocytes are under redox control [53]. Although the presence of a high iron concentration, hemoglobin, and both non-enzymatic and enzymatic antioxidants provide erythrocytes with an antioxidant capacity, erythrocytes can become the target of xenobiotics or ROS produced by nearby tissues, thus accumulating oxidative damage and then being removed. Several components of the apoptotic machinery found in nucleated cells were identified to be upregulated in mammalian erythrocytes under oxidative stress. Procaspase 3 was found in mature human erythrocytes and is activated under oxidative stress upon *t*-butylhydroperoxide treatment leading to impairment of aminophospholipid flippase activity, phosphatidylserine externalization, and increased erythrophagocytosis [54]. Components of the extrinsic apoptotic pathway including FasL, the Fas-associated death domain, and caspase 8 were shown to form complexes in membrane rafts of aged and oxidatively stressed erythrocytes. A previous study also showed that oxidative stress can activate Ca^2+^-permeable cation channels, cause increased cytosolic calcium concentrations, and trigger erythrocyte apoptosis [55].

### Induction of cell death of erythrocytes and the endocardium in zebrafish embryos by β-lapachone is mediated through an NQO1- and calcium-dependent pathway

The linear arrangement of the ventricle and atrium and the presence of only a few or no erythrocytes in circulation were observed in β-lapachone-treated 52-, 72-, and 96-hpf embryos (Figure 1). In addition, β-lapachone treatment caused the occurrence of ROS in erythrocytes and DNA fragmentation in erythrocytes and the endocardium of zebrafish embryos (Figure 3). In a number of tumors which possess high expression levels of NQO1, β-lapachone can be reduced by NQO1 and leads to a futile cycle between quinone and hydroquinone, accompanied by depletion of NAD(P)H and the generation of ROS. ROS can cause DNA damage, hyperactivation of PARP-1, which depletes NAD^+ ^and ATP pools that results in increased intracellular cytosolic Ca^2+ ^concentrations, and the activation of μ-calpain cysteine protease activity [23-25]. We also showed that both the heart-looping defect and erythrocyte-deficiency in circulation phenotypes could be rescued by co-incubation of β-lapachone with either dicoumarol, an NQO1 inhibitor, or BAPTA-AM, a Ca^2+ ^chelator (Figure 5). These results indicate that the induction of cell death of erythrocytes and the endocardium in zebrafish embryos by β-lapachone is mediated through an NQO1- and calcium-dependent pathway.

A recent study also showed that oral treatment of rats with β-lapachone induced anemia that was attributed to increased phosphatidylserine externalization and erythrophagocytosis induced by ROS [56]. However, the extents of reduction in erythrocyte cell numbers in adult rats (22%~26%) and zebrafish embryos (75%~90%) upon β-lapachone treatment greatly differed. This discrepancy may have been due to the presence of mitochondria in mature fish erythrocytes but not in mature mammalian erythrocytes [57]. It was shown that over 60 moles of NAD(P)H are used by NQO1 to react with each mole of β-lapachone. Since the citric acid cycle in mitochondria can produce more NADH than glycolysis in the cytosol, we suggest that more ROS can be generated in zebrafish erythrocytes than in rat erythrocytes upon β-lapachone treatment. ROS then cause DNA damage, an increased cytosolic Ca^2+ ^concentration either from endoplasmic reticulum release or entry from Ca^2+^-permeable cation channels, loss of ATP and NAD^+^, and inhibition of DNA repair, leading to cell death [23].

## Conclusions

Our study indicates that β-lapachone treatment causes death of the endocardium and erythrocytes in zebrafish embryos resulting in defects in heart-looping, valve formation, and cardiac output. The induction of apoptosis of the endocardium and erythrocytes by β-lapachone is mediated through an NQO1- and calcium-dependent pathway. This study further demonstrated the importance of the endocardium and hemodynamic forces to heart morphogenesis and contractile performance.

## List of abbreviations used

hpf: hours post fertilization; DMSO: dimethyl sulfoxide; ODA: *o*-Dianisidine; NQO1: NAD(P)H:quinone oxidoreductase; PARP-1: poly(ADP-ribose) polymerase-1; FS: fractional shortening; VB: ventriculobulbal; AV: atrioventricular; DIC: differential interference contrast; TRITC: tetramethylrhodamine isothiocyanate

## Competing interests

The authors declare that they have no competing interests.

## Authors' contributions

YTW conducted the drug treatment, whole-mount *in situ *hybridization, histological sectioning, TUNEL assay, and interpretation of data. LCY participated in the ROS, fractional shortening, rhodamine dye injection, and wall shear stress measurement. MYT carried out the whole-mount *in situ *hybridization and interpretation of data. YHC participated in the initial drug screening and histological sectioning. YFL carried out the histological sectioning and TUNEL assay. CJH participated in the contribution of reagents and interpretation of data. CMC participated in the interpretation of data and helped to draft the manuscript. SPLH participated in the design of this study, performed statistical analysis, interpretation of data and draft the manuscript. All authors read and approved the final manuscript.

## Supplementary Material

Additional file 1 **Figure S1 Effects of β-lapachone treatment on the expressions of *c-myb *and *ikaros***. Embryos were treated with DMSO or β-lapachone at 24 hours post-fertilization (hpf) for 4 h and fixed at 30 hpf for whole-mount *in situ *hybridization using either *c-myb *(A, B) or *ikaros *(C, D) as RNA probes. Scale bars represent 100 μm. Click here for file
