# Supplementary figures and images for: β-Lapachone induces heart morphogenetic and functional defects by promoting the death of erythrocytes and the endocardium in zebrafish embryos
Source: J Biomed Sci. 2011 Sep 22;18(1):70. doi: 10.1186/1423-0127-18-70 (PMC3197495; doi:10.1186/1423-0127-18-70)

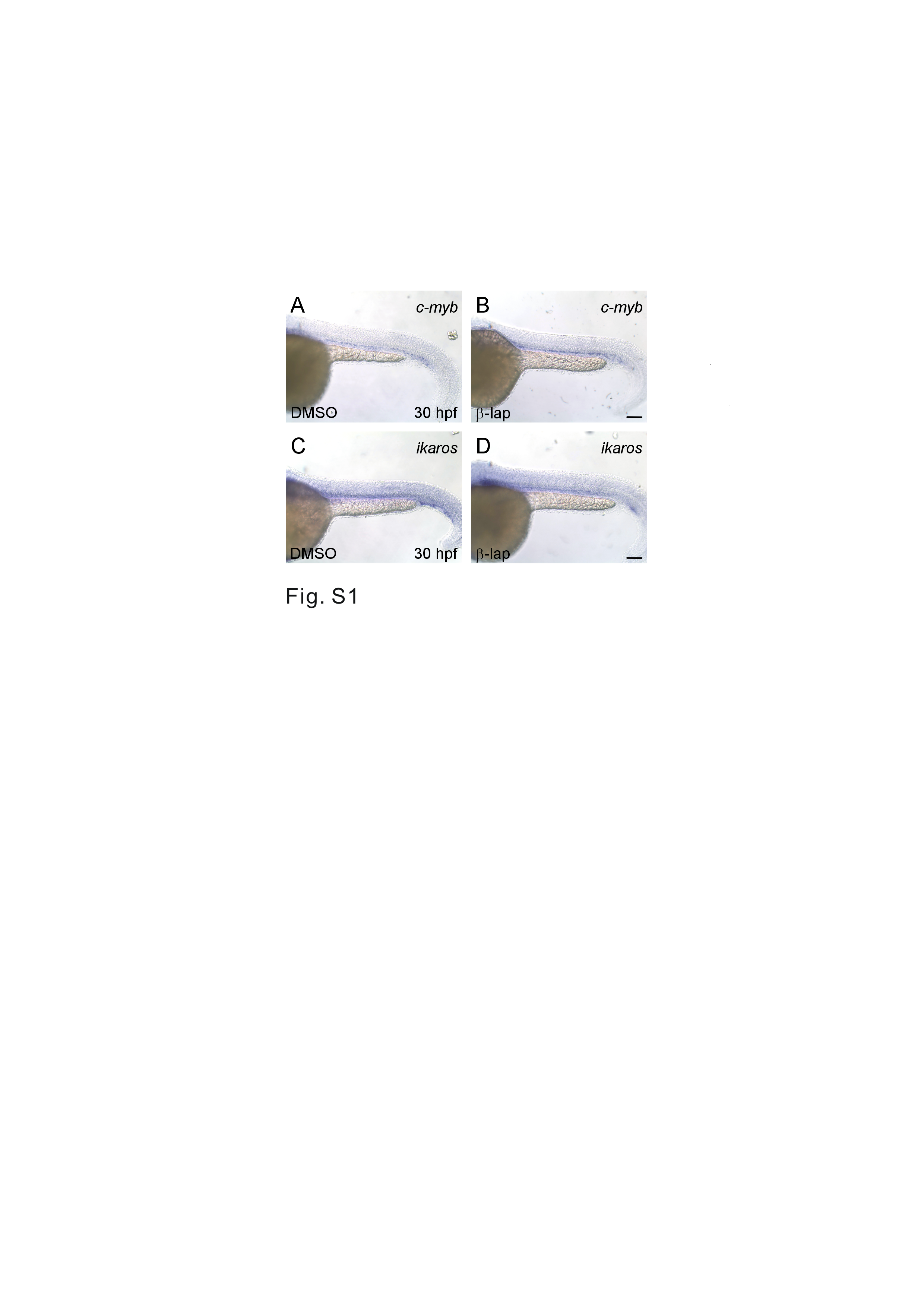

Supplement: Additional file 1 — Figure S1 Effects of β-lapachone treatment on the expressions of c-myb and ikaros . Embryos were treated with DMSO or β-lapachone at 24 hours post-fertilization (hpf) for 4 h and fixed at 30 hpf for whole-mount in situ hybridization using either c-myb (A, B) or ikaros (C, D) as RNA probes. Scale bars represent 100 μm. [file 1423-0127-18-70-S1.TIFF]
